# Psychometric validation of a novel community norms measure among youth, Eswatini Violence Against Children and Youth Survey, 2022

**DOI:** 10.1371/journal.pone.0345048

**Published:** 2026-05-20

**Authors:** Stephanie Spaid Miedema, Greta M. Massetti, Francis B. Annor, Laura F. Chiang, Rebecka Lundgren, Anita Raj

**Affiliations:** 1 Division of Violence Prevention, National Center for Injury Prevention and Control, Centers for Disease Control and Prevention, Atlanta, Georgia, United States of America; 2 School of Public Health, Georgia State University, Atlanta, Georgia, United States of America; 3 Center on Gender Equity and Health, University of California San Diego, San Diego, California, United States of America; 4 Newcomb Institute, Tulane University, New Orleans, Louisiana, United States of America; 5 Celia Scott Weatherhead School of Public Health and Tropical Medicine, Tulane University, New Orleans, Louisiana, United States of America; PLOS: Public Library of Science, UNITED KINGDOM OF GREAT BRITAIN AND NORTHERN IRELAND

## Abstract

Social norms define what is acceptable and appropriate for women and men, boys and girls, in a given group or society. Restrictive social norms around women and men’s roles and responsibilities have proven harmful for both women and men, particularly in adolescence and young adulthood, and are associated with increased risk of violence. In global settings, measurement of social norms tends to rely on proxy measures capturing attitudes, beliefs, and perspectives. Measurement of social norms on women’s and men’s roles and responsibilities is particularly limited among adolescents and young adults, a formative age period where sanctions for non-adherence to norms can be heavy. We used data from the nationally representative 2022 Eswatini Violence Against Children and Youth Survey (VACS) to test the psychometric properties of a novel set of social norms survey items among male and female youth aged 13−24 (n = 7,709). Items were largely derived from prior scales and adapted by social norms experts to ensure that they were salient to the lives of adolescents in low-income settings. The items captured norms about women and men’s, boys’ and girls’ education, domestic labor, household decision-making, work, marriage and violence, with the community as the reference group. We conducted exploratory (EFA) and confirmatory factor analysis (CFA) on 16 norms survey items using split-random half samples. We identified a single-factor, four-item scale as the best-fitting solution (EFA: RMSEA = 0.060, CFI = 0.974, TLI = 0.923, SRMR = 0.076; CFA: RMSEA = 0.063, CFI = 0.972; TLI = 0.915; SRMR = 0.048). The scale captured norms in communities on domestic labor for youth and household-decision making by adults. We assessed measurement invariance by age and sex of this final one-factor, four-item solution. We observed latent mean differences by sex in baseline (β = 0.309, *p* < 0.001) and final DIF-adjusted (β = 0.299, *p* < 0.001) models. In addition, females and males varied in their propensity to endorse two of the scale items (Item 5: β = 0.225 *p* < 0.001; Item 6: β = −0.212, *p <* 0.001). We assessed correlation between the final DIF-adjusted community norms measure and two attitude scales: attitudes toward IPV (*r* = 0.363, *p* < 0.001) and attitudes toward women and men’s relations (*r* = 0.087, *p* = 0.018). The measure demonstrated adequate reliability and convergent validity. The analysis resulted in a promising single factor, four-item social norms scale, which meets requirements of a brief, theory-based measure and is feasible to incorporate into national and cross-national surveys. Future work is needed to validate the scale in other settings.

## Introduction

Social norms are the informal rules and expectations that govern and regulate behavior in groups or societies imparted via socialization across the life course [1–3]. These norms are socially defined and specify what behaviors are proper and acceptable versus improper and unacceptable [4]. They have the purpose of maintaining social order and control, and they are important in the basic functioning of any social grouping [5,6]. Social norms include both a) descriptive norms: what a person thinks others in their given social group are doing or not doing and b) injunctive norms: the person’s perception of what others in their given reference group think they should or should not do. They are defined, enforced, and reinforced in day-to-day aspects of life via social sanctions, which we define as the social reaction of approval or disapproval – rewards or punishments – to one’s behaviors [7,8].

### Norms around women’s and men’s roles, behaviors and responsibilities

Growing evidence and investments have focused on norms around women and men’s roles, behaviors and responsibilities in society as a means of improving health and economic development and preventing violence [9–12]. These social norms in particular define what are acceptable and appropriate roles, responsibilities and relations for women and men, boys, and girls, in each group or society. Restrictive social norms have proven harmful for both women and men, particularly in adolescence and young adulthood [10]. To date, lack of quantitative data at scale on social norms impedes our ability to examine this issue, link it directly with outcomes of interest, and track its change over time, particularly as it relates to evaluation of social norm intervention efforts [13–15]. Currently, research largely relies on attitudinal rather than norm measures, as seen for example in UNDP’s 2023 Gender Norms Index [16]. This measure involves asking individuals about their personal beliefs and perspectives on issues. For example, items ask whether the respondent believes that “men make better political leaders than women do” and “men should have more right to a job than women.” Consistently, measures of inequitable attitudes have been associated with harmful social behaviors, such as increased risk of violence against children and women [17–19]. Thus, such measures can be useful, and often are indirectly informed by broader social norms, but they do not directly measure social norms, which would instead focus on the expectations and beliefs in a community and society, rather than those of an individual [10]. Overall, there is growing consensus around the importance of adequately measuring norms – both descriptive and injunctive, rather than relying on attitudes and beliefs as proxies for social expectations [20–22].

There are few available theoretically grounded measures that capture social norms around women and men’s roles, responsibilities and relations in society [20]. Measures often fail to differentiate between descriptive and injunctive norms and often do not include a specific reference group [22,23]. However, progress is being made in this area. For example, a measure of injunctive and descriptive social norms applied among adolescents and young adults in a site-specific study in Kinshasa included nine items for family planning use and eight for relations between women and men, girls and boys [24]. A reliable norms measure used with adolescents ages 10–12 in sites in Bangladesh and Ethiopia consists of 14 questions assessing injunctive and descriptive norms [25]. Another notable theory-based norms scale, the G-NORM scale, with high internal validity is gaining traction [20]. It has two subscales, with one factor each, comprising nine items representing the overall construct of descriptive and injunctive social norms. Yet, despite the high quality of these scales, their length requires considerable real estate and may preclude inclusion in population-based surveys.

### Social norms in adolescence

Adolescence is a time when norm formation starts to occur, with heavier sanctioning for non-adherence to social norms, particularly those related to women and men’s roles, behaviors and responsibilities in society [7]. Unfortunately, there is even less research on norms of adolescents captured by large-scale population surveys than that seen for adults, again limiting our understanding of this issue. The strongest population-level data that captures social norms with an adolescent population is from the Global Early Adolescent Study. In this study, norms were measured as adolescent perceptions regarding what were normative expectations of girls and boys in their community regarding: a) sexual double standards (e.g., male romantic insincerity, female sexual gatekeeping), b) stereotypic traits for girls and boys (e.g., male toughness, female humility), and c) stereotypic roles for women and men (e.g., male breadwinning, female caregiving) [26]. Findings from this work document substantial variability across nations and among adolescents. However, the Global Early Adolescent Study did not yield an overall social norms measure around roles, behaviors and responsibilities for girls and boys, women and men, although research in Kinshasa and Hanoi did produce a valid scale measuring norm around intimate relationships among young adolescents including “Adolescent Romantic Expectations” and “Sexual Double Standard” subscales [ 15]. In sum, we lack theoretically grounded, well-validated and parsimonious social norms measures on women’s and men’s, girls’ and boys’ roles, responsibilities and behaviors among adolescents that can be implemented through population-level surveys.

### Measurement challenges and opportunities

As alluded to above, unique challenges to measurement of social norms exist. By definition, norms are embedded at community or societal levels, making them difficult to measure through questions posed to individual survey respondents. Norms can be measured using the approach best suited to respondents such as single items, scales, or indices as long as they refer to specific behaviors in a concrete scenario [21,22,24,27]. Because norms are based on social processes and structures, measuring them often requires multiple questions about different domains of a norm and corresponding behaviors [24]. Social norm theorists recommend measuring perceptions of typical behavior (descriptive norms), social approval (injunctive norms) or, ideally, both [23]. Understanding social norms and their influence on behavior requires measures that address the strength of the norm, determine whether sanctions maintaining the norm exist and assess how sensitive individuals are to them [28,29]. There is emerging consensus that norms measures must refer to the reference group –those who influence compliance with social expectations. Asking questions about behaviors or beliefs without asking about the reference group will result in a measure of an individual’s attitude or personal belief, not a social norm [30].

In sum, the science of norms measurement on household surveys, particularly for youth, is limited and the field lacks consensus on the gold standard approaches to measuring norms related to women and men’s roles, behaviors and responsibilities in society and how these norms relate to health and behavioral outcomes. This highlights the need for research to identify measures of norms and validate them using large scale national surveys with adolescents and young adults in diverse geographical and cultural settings. A validated measure that assesses social norms prevalent in the communities where youth live has potential to address gaps in measurement and monitoring of social norms at population levels among youth in LMICs.

A variety of data collection methods have been explored to measure social and community norms, as well as assess the relationship between norms and behavioral and health outcomes. National household surveys, such as the Violence Against Children and Youth Surveys (VACS), provide a valuable platform to assess and monitor population-level changes in social norms among adolescents, and associations between social norms and adolescent health and behavioral outcomes. In 2022, the Eswatini VACS included novel items to measure and monitor population-level social norms among youth related to domains salient to the lives of youth, such as education, domestic labor, household decision-making, work, marriage and violence [31–33]. As the norms measures used youths’ communities as a reference group, we here on refer to them as community norms).

Eswatini (formerly Swaziland) serves as a valuable national context for testing a novel social norms measure among adolescents in global contexts. Eswatini is a landlocked country of slightly over 1 million people, bordering Mozambique and South Africa. The country is predominantly rural and low-income. It is largely homogenous in terms of ethnicity, religious practice, social expectations, and income, enabling measurement invariance testing of this new community norms measure by age and sex with limited risk of confounding by other social factors.

The purpose of the present study was to assess the psychometric validity of this new community norms measure, testing it with a generalizable sample of youth in a single country context, to determine its potential value of use at scale. Specifically, we seek to assess the latent factor structure of the new community norms measure, evaluate the internal consistency of this novel measure of community norms by sex and age, and confirm the construct validity of the final identified measure.

## Methods

### Data & participants

**Fig 1:**
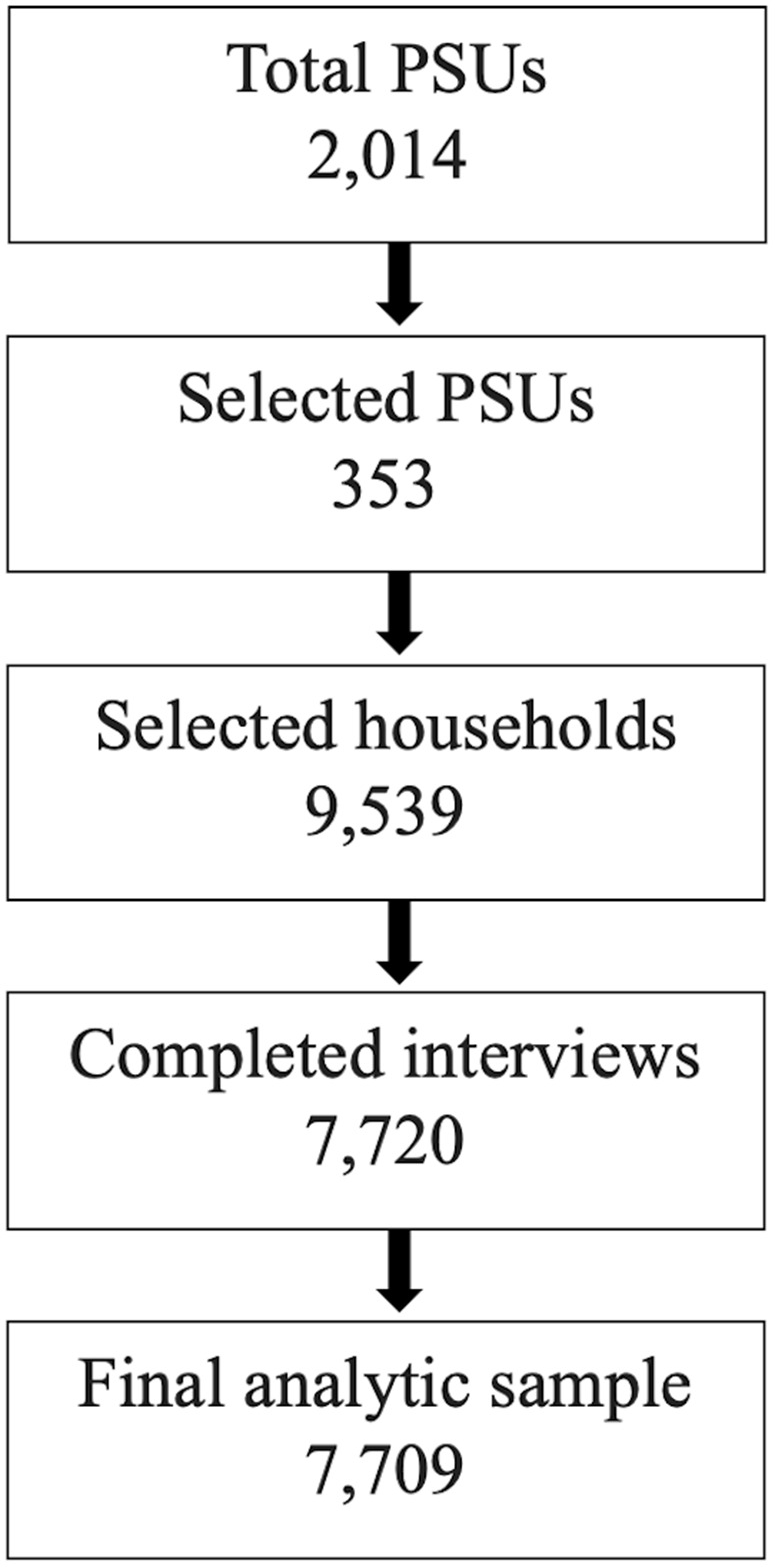
Sampling and population selection of the 2022 Eswatini Violence Against Children Survey community norms analytic sample.

Data were derived from the 2022 Eswatini VACS. The data was made available to CDC by the Government of Eswatini through a data-sharing agreement, and data were accessed for research purposes on 31/10/2022. Representative samples for males in the 2022 Eswatini VACS were drawn at the national level only. For females, representative samples were drawn for each region. In addition, for females, each of the four regions were oversampled where the PEPFAR DREAMS HIV prevention program for adolescent girls and young women was implemented [34,35]. The 2022 Eswatini VACS sampling frame was based on the 2017 Eswatini Population and Household Census. A split sampling approach was used for the 2022 Eswatini VACS such that separate PSUs were sampled for males and females, to ensure that males and females were not interviewed in the same area. In each selected PSU, households were pre-screened for eligible participants. During the mapping and listing of household structures in each PSU, field staff determined if there was an eligible participant in that household. A random selection of pre-screened households was selected within each PSUs, and an eligible 13–24-year-old male or female participant was selected per household. To ensure participant safety and reduce risk that other household members would know the survey contents, only one participant was selected per household [36,37]. The overall response rates were 90.1% for females and 84.7% for males. The 2022 Eswatini VACS received independent ethical review and approval from the Institutional Review Boards for CDC, Columbia University, and the Eswatini Health and Human Research Review Board. A total of 7,720 youth aged 13–24 completed interviews (n_f_ = 6,318; n_m_ = 1,402). Descriptive statistics for the full sample is available in S1 Table. Eleven observations had missing responses for all 17 items included in the community norms measure and were dropped from the analytic sample. The final analytic sample was 7,709 youth. See Fig 1 for details on sampling and sample selection for the present analysis.

### Measures

#### Norms.

The 2022 Eswatini VACS included 14 items measuring community norms on education, domestic labor, household decision-making, work and marriage selected from previously tested measures [25,38], and 3 novel norms measures related to family violence developed specifically for VACS. A multidisciplinary team of social norms experts selected and adapted items from existing measures for use in VACS contexts. Selected items assessed both descriptive norms (what the participant thinks people in their community do) and injunctive norms (what the participant thinks their community expects people to do) related to the following domains: (1) prioritization of boy’s education relative to girls’ education, (2) girl’s relative to boy’s responsibility for domestic labor, (3) early marriage of girls, (4) parents’ control of the behavior of girls relative to boys, (5) men’s control over household decision-making, and (6) women’s participation in work outside the home. Three additional violence-related norms items were developed by the team of experts. The three violence-related norms included the following questions: “of the married men, or men in relationships in your community, how many would you say hit their wives or girlfriends/romantic partners to correct them when they feel the wife/girlfriend/romantic partner has done something wrong?”; “most adults in my community intervene, for example talk to the family or call police or other authorities, if their neighbor is hitting, or intentionally hurting, his wife”; “most adults in my community intervene, for example, talk to the family or call police or other authorities if their neighbor is hitting, or intentionally hurting, their child. Overall, all scale items were selected based on a priori assessment of social arenas salient to the lives of adolescents, and particularly girls and young women, in low-income contexts, including Eswatini [31,32,39].

Likert scale responses to all but one item were: strongly agree, agree, neither agree/disagree, disagree, strongly disagree, don’t know, declined to answer. To facilitate interpretation of factor analytic results, response options were merged to create a three-category variable: strongly agree/agree, neither agree/disagree, disagree/strongly disagree. Items were coded so that 1 = equal/positive responses, 2 = neutral responses, and 3 = not equal/harmful responses. For example, for item 1 (“our culture makes it harder for girls to achieve their goals than boys”), those responding disagree or strongly disagree would be indicating the presence of more equal norms between boys and girls in their community, that is, they perceive that their culture *does not* make it harder for girls to achieve their goals relative to boys. For this item, the disagree/strongly disagree response category was coded 1 to indicate equal/positive responses. Conversely, for the item “women in my community work outside the home”, those endorsing the agree/strongly agree response options would be indicating the presence of a more equal community for women and men. For this item, agree/strongly agree was coded 1 to indicate equal/positive responses.

A single item (“Of the married men or men in relationships in your community, how many would you say hit their wives or girlfriends/romantic partners to correct them when they feel the wife/girlfriend/romantic partner has done something wrong?”) had different response options from the remaining items. Response options included: almost all the men in the community, more than half, less than half, a few, no men, don’t know, declined. Furthermore, over 40% of the participants responded “Don’t Know” to this item. Due to the large amount of missingness, and its unique response categories, this item was dropped from analysis.

We assessed a final set of 16 items (S1 Table). In order to understand the quality of the measures, don’t know and declined to answer responses were assessed, along with variability in responses, for all items (S2 Table). For purposes of measurement testing, Don’t Know and Declined to Answer responses were set to missing.

#### Attitude scales.

To assess convergent validity of the final scale, we used the Demographic and Health Survey (DHS) attitudes toward intimate partner violence (IPV) scale, and the VACS attitudes toward women and men’s relations scale. The DHS scale is widely used and validated [40]. Youth were asked whether they believed a husband was justified in hitting or beating his wife under a series of conditions. Cronbach’s alpha for the five items was 0.842. Youth who agreed with at least one condition under which they believed intimate partner violence is justifiable were coded as 1. Youth who did not agree that any condition was justifiable were coded as 0. The VACS attitudes toward women and men’s relations scale has historically been used as a proxy for social norms in analyses with VACS data [17]. Youth were asked a series of 5 questions on women and men’s roles and responsibilities with respect to sex, reputation, and violence. Youth who agreed with at least one question were coded as 1. Youth who disagreed with all five questions were coded as 0.

### Data analysis

#### Exploratory and confirmatory factor analysis.

We conducted exploratory (EFA) and confirmatory (CFA) analysis on the final 16 community norms items using the full sample of male and female youth. We used a random number generator to create random split-half samples. Factor analyses were adjusted for sample weight and used variance-adjusted weighted least squares (WLSMV) estimation in Mplus software [41,42]. We first ran EFA to determine the underlying factor structure of this novel set of community norms items [43]. For EFA, we ran sequential 1- through 7-factor models to test alternative solutions, with geomin rotation [41]. Model fit was evaluated based on RMSEA (<0.05), TLI (>0.90), CFI (>0.90) and SRMR (<0.08) fit indices [44]. A priori selection criteria included dropping items that loaded <0.4 or cross-loaded at>=0.4. If fewer than 3 items loaded onto a single factor, those items were considered inadequate for measurement purposes and were dropped [45]. Using these a priori selection criteria, the team selected the most parsimonious solution.

We took a series of steps to assess the effect of missingness (inclusive of don’t know/declined to answer responses) on EFA and concluded that missingness did not influence the selection of a final best-fitting solution. Among the 16 items, missingness ranged from 4.69% (item 3) to 32% (item 16). Analysis of missingness on the two variables with the highest levels of missingness (item 15 and item 16) was conducted. Females and youth aged 13–17 were significantly more likely to be missing on both items. Results are available upon request. Sensitivity analyses were conducted to assess the effect of missingness on EFA. First, we identified indicators for which missingness was > 10%. Then we dropped any observation missing on all of these indicators and re-ran sequential EFA on the smaller data set. Second, we re-ran sequential EFA on the complete case data set (approximately 35% of the original data set). We observed no substantive differences in the EFA factor loading patterns, and so continued analysis with the full sample of 7,709 females and males. We then estimated confirmatory factor solutions on the second random split sample for the best-fitting model identified through EFA, using the above-described fit indices.

#### Measurement invariance.

Measurement invariance exists if the distribution of the item responses depends only on the individual’s latent variable values, and not on other individual characteristics, such as age or sex [46]. We used single-group Multiple Indicator Multiple Cause (MIMIC) structural equation models to assess invariance of indicator intercepts and factor means by age and sex. MIMIC models are CFA models with covariates. The inclusion of direct effects of covariates with factor indicators can help to establish measurement invariance or differential item functioning (DIF) of indicators of a latent factor [42]. DIF occurs if individual from two groups with equivalent levels of a latent construct have differential probability of selecting responses for construct indicators. Groups are differentiated via use of the exogenous covariate. We added sex and age as exogenous covariates to best-fitting solution for the baseline MIMIC model. We assessed modification indices to identify parameters that could be estimated to improve model fit. We made changes to model specification where MI indicated DIF by age or sex > 10.0 (i.e., the groups respond differently to that item).

#### Reliability and convergent validity.

We used Cronbach’s alpha to assess scale reliability (internal consistency). To assess scale convergent validity (construct validity), we examined correlations between the final DIF-adjusted MIMIC model and the DHS measure of attitudes condoning IPV and the attitudes toward women’s and men’s relations, included in the VACS. The attitudes scales both demonstrated strong unidimensional structure (results available upon request).

## Results

### Descriptives

Table 1 presents weighted descriptive results for the 16 items measuring community norms in the 2022 Eswatini VACS. Over three in four (77.88%) participants endorsed positive/equal responses to item 3 (“Girls in my community are sent to school only if they are not needed to help at home”). In other words, the majority of youth disagreed or strongly disagreed with this item. Conversely, 76.29% of participants endorsed a negative/unequal response to item 8 (“Most men in my community are the ones who make the decisions in their home”). For this item, most youth agreed or strongly agreed with the item. Items tended to be skewed toward one type of response (i.e., more positive/equal or more negative/unequal). However, a few items were more evenly distributed across the sample. For example, 43.01% of youth felt that their culture makes it harder for girls to achieve their goals than boys, while 36.56% of youth felt the opposite.

**Table 1:** Weighted descriptives of items measuring community norms among youth ages 13-24 years, 2022 Eswatini Violence Against Children and Youth Survey (n = 7,709).

Item	Item Description		Response Type*		
			Equal community	Neutral	Unequal community
Item 1	Our culture makes it harder for girls to achieve their goals than boys	n	2,858	717	3,396
		%	36.56	11.54	43.01
Item 2	Adolescent girls in my community are more likely to be out of school than adolescent boys	n	4,214	667	2,415
		%	53.07	9.84	32.58
Item 3	Girls in my community are sent to school only if they are not needed to help at home	n	6,235	492	631
		%	77.88	7.43	10.66
Item 4	Most people in my community expect girls to be sent to school only if they are not needed at home	n	6,199	486	656
		%	77.34	7.42	11.00
Item 5	Most boys and girls in my community do not share household tasks equally, with girls doing more household tasks than boys	n	2,108	484	4,668
		%	31.45	7.64	55.01
Item 6	Most people in my community expect men to have the final word about decisions in the home	n	1,673	519	5,010
		%	20.94	8.08	65.26
Item 7	Most people in my community do not expect girls and boys to share household tasks equally because they expect girls to do more in the household	n	2,593	575	4,018
		%	36.74	8.60	47.90
Item 8	Most men in my community are the ones who make the decisions in their home	n	906	453	5,863
		%	11.55	6.96	76.29
Item 9	Women in my community work outside the home	n	4,949	458	1,350
		%	63.09	6.42	21.31
Item 10	Most people in my community believe that women should be able to work outside the home if they want	n	4,770	547	1,271
		%	61.37	8.77	17.69
Item 11	Most adolescent girls in my community marry before the age of 18 years	n	5,784	374	847
		%	73.80	5.06	12.17
Item 12	Adults in my community expect adolescent girls to get married before 18 years	n	5,924	431	577
		%	75.07	6.26	8.79
Item 13	Most families in my community control their daughters’ behaviors more than their sons’	n	1,529	500	5,056
		%	19.93	7.80	65.41
Item 14	Most people in my community expect families to control their daughter’s behavior more than their sons’	n	1,143	481	5,473
		%	15.78	8.09	69.56
Item 15	Most adults in my community intervene, for example, talk to the family or call police or other authorities, if their neighbor is hitting or intentionally hurting, his wife.	n	4,047	369	805
		%	54.27	4.88	10.26
Item 16	Most adults in my community intervene, for example, talk to the family or call police or other authorities, if their neighbor is hitting or intentionally hurting, their child.	n	4,021	345	1,026
		%	53.74	5.06	14.68

*Equal community responses captured “strongly agree/agree” response options if the statement described a community environment that was more equal for boys and girls, women and men, and “strongly disagree/disagree” response options if the statement described a community environment that was not equal between boys and girls, women and men. Neutral responses captured “neither agree nor disagree” response options. Unequal community responses captured “strongly agree/agree” response options if the statement described a community environment that was not equal between boys and girls, women and men, and “strongly disagree/disagree” response options if the statement described a community environment that was equal between boys and girls, women and men.

### Exploratory and confirmatory factor analysis

Sequential EFA identified a six-factor solution as the best-fitting solution. However, four of the six identified factors included only two loaded items each, which was inadequate to measure the underlying constructs. As such, these items were dropped. This initially left us with a two-factor, seven-item solution, inclusive of items 2–4 (Factor 1) and items 5–9 (Factor 2). However, further investigation identified a negative residual variance for item 3 for both 13–17-year-olds and females and so item 3 was removed from analysis. As Factor 1 was left with only two items, we removed Factor 1 from future analyses. EFA and CFA results for the 2-factor, 7-item solution is available in S2 Table. A 1-factor, 4-item factor structure was identified as the best-fitting and most parsimonious solution (Table 2). Factor loadings ranged from 0.672 (“Most boys and girls in my community do not share household tasks equally, with girls doing more household tasks than boys”) to 0.792 (“Most people in my community expect men to have the final word about decisions in the home”). Fit statistics demonstrated adequate fit: RMSEA = 0.060, CFI = 0.974, TLI = 0.923, SRMR = 0.076, although the RMSEA statistic was slightly elevated above the threshold of 0.05. The factor solution captures community norms related to household roles and responsibilities.

**Table 2:** Results of exploratory and confirmatory factor analysis of items measuring domestic labor and household decision-making community norms among youth ages 13-24 years, 2022 Eswatini Violence Against Children and Youth Survey.

	EFA (n = 3,842)^±^	CFA (n = 3,827)^±^		
	Loading	Estimate	S.E.	*p*
Most boys and girls in my community do not share household tasks equally, with girls doing more household tasks than boys (Item 5)	0.672*	0.696	0.029	<0.001
Most people in my community expect men to have the final word about decisions in the home (Item 6)	0.792*	0.775	0.028	<0.001
Most people in my community do not expect girls and boys to share household tasks equally because they expect girls to do more in the household (Item 7)	0.758*	0.747	0.024	<0.001
Most men in my community are the ones who make the decisions in their home (Item 8)	0.692*	0.755	0.033	<0.001
RMSEA (90% Confidence Interval)	0.060 (0.042, 0.080)	0.063 (0.045, 0.083)		
CFI	0.974	0.972		
TLI	0.923	0.915		
SRMR	0.076	0.048		

*Significant at the.05 level. ^**±**^The full analytic sample (n = 7,709) was randomly split into two halves for EFA and CFA validation.

CFA confirmed the one-factor solution identified through EFA (Table 2). For CFA, factor loadings were strong and ranged from 0.696 (“Most boys and girls in my community do not share household tasks equally, with girls doing more household tasks than boys”) to 0.775 (“Most people in my community expect men to have the final word about decisions in the home”). CFA fit statistics for the identified solution had adequate fit: RMSEA = 0.063, CFI = 0.972, TLI = 0.915, SRMR = 0.048, although, similar to the EFA results, the RMSEA statistic was slightly elevated above the recommended threshold.

### Measurement invariance

Table 3 provides single-group models for the 4-item, 1-factor solution by age and sex. We observed the good fit statistics for all sub-groups, although fit statistics were lowest among the male single-group solution (RMSEA = 0.087, CFI = 0.964, TLI = 0.893).

**Table 3:** Single-group models for domestic labor and household decision-making community norms single factor 4-item solution by age and sex, 2022 Eswatini Violence Against Children and Youth Survey.

Single-group models	n	WLSMV ↓2 (p)	df	RMSEA [95% CI]	CFI	TLI
Female	6196	43.273 (<0.001)	2	0.058 [0.044, 0.073]	0.983	0.950
Male	1369	22.935 (<0.001)	2	0.087 [0.058, 0.121]	0.964	0.893
13-17	3697	32.473 (<0.001)	2	0.064 [0.046, 0.084]	0.970	0.911
18-24	3868	36.141 (<0.001)	2	0.066 [0.049, 0.086]	0.977	0.930

WLSMV = Weighted Least Squares Mean and Variance adjusted; df = degrees of freedom; RMSEA = Root Mean Square Error of Approximation; CFI: Comparative Fit Index; TLI: Tucker Lewis Index.

**Fig 2:**
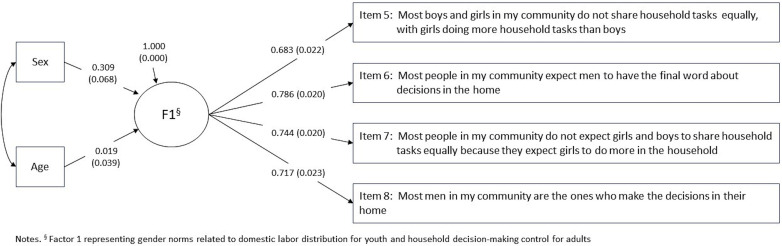
Beta estimates and standard errors of baseline Multiple Indicator Multiple Cause model for four-item single factor measure of domestic labor and household decision-making community norms, by sex and age, among youth aged 13-24, 2022 Eswatini Violence Against Children and Youth Survey.

**Fig 3:**
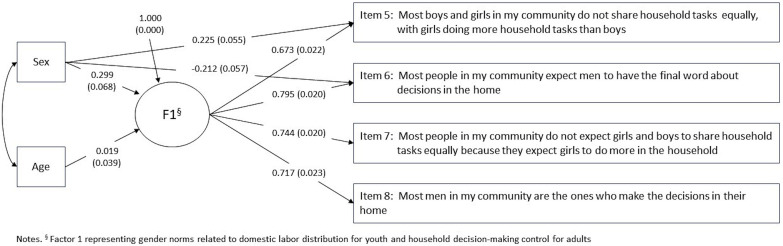
Beta estimates and standard errors of Multiple Indicator Multiple Cause model adjusted for Differential Item Functioning for a four-item single factor measure of domestic labor and household decision-making community norms, by sex and age, among youth aged 13-24, 2022 Eswatini Violence Against Children and Youth Survey.

To test for measurement invariance, we added sex and age as exogenous covariates to the 4-item, 1-factor CFA solution for the baseline MIMIC model and evaluated modification indices. Fig 2 and Table 4 present results from the baseline MIMIC model. We observed a statistically significant direct effect between sex and the latent factor (β = .309, *p* < 0.001) in the baseline model, indicating latent mean differences across the groups. Modification indices from the baseline MIMIC model indicated DIF by sex for two indicators: item 5 (16.508) and item 6 (13.563), so we estimated direct effects between sex and each indicator (Fig 3 and Table 4). Direct effects between sex and item 5 (β = .225, *p* < 0.001) and item 6 (β = −.212, *p* < 0.001) were statistically significant, indicating that females had a greater propensity (compared to males) to endorse item 5 and a lesser propensity (compared to males) to endorse item 6, holding constant their levels of the underlying construct. Girls and boys may understand items differently or may be differentially motivated to select certain response categories, even when they share similar overall views of women and men’s roles in society. Fit statistics were slightly improved from the baseline model.

**Table 4:** Baseline and DIF-adjusted MIMIC models of domestic labor and household decision-making community norms by sex and age, 2022 Eswatini Violence Against Children and Youth Survey (n = 7,565).

	Baseline		Adjustment for DIF					
	DIF (item 5)		DIF (item 6)		DIF (item 5,6)	
Factor Indicators	Est	*p*	Est	*p*	Est	*p*	Est	*p*
Item 5	0.683	<0.001	0.674	<0.001	0.682	<0.001	0.673	<0.001
Item 6	0.786	<0.001	0.791	<0.001	0.795	<0.001	0.795	<0.001
Item 7	0.744	<0.001	0.746	<0.001	0.741	<0.001	0.744	<0.001
Item 8	0.717	<0.001	0.719	<0.001	0.712	<0.001	0.716	<0.001
**Direct Effects (DIF)**								
Item 5 on sex			0.278	<0.001			0.225	<0.001
Item 6 on sex					−0.288	<0.001	−0.212	<0.001
**Structural Regressions**								
Sex (ref = male)	0.309	<0.001	0.220	0.001	0.392	<0.001	0.299	<0.001
Age (ref = 13–17)	0.019	0.619	0.019	0.626	0.019	0.627	0.019	0.632
**Model Fit Statistics**								
RMSEA (90% CI)	0.037 (0.030, 0.044)		0.035 (0.028, 0.043)		0.036 (0.029, 0.043)		0.035 (0.028, 0.044)	
CFI	0.958		0.966		0.965		0.970	
TLI	0.927		0.932		0.930		0.931	

DIF = Differential Item Functioning; MIMIC = Multiple Indicator Multiple Cause; RMSEA = Root Mean Square Error of Approximation; CI = Confidence Interval; CFI: Comparative Fit Index; TLI: Tucker Lewis Index.

### Reliability and validity

Cronbach’s Alpha for the 4-item scale was 0.7706, which demonstrates acceptable internal consistency [47]. We assessed correlations between the DIF-adjusted community norms scale and two attitudinal scales from VACS: the attitudes condoning IPV scale and the attitudes toward women and men’s relations scale. The relationship between the DIF-adjusted community norms scale and the IPV attitudes scale yielded a moderate correlation (*r* = 0.363, *p* < 0.001), suggesting that the two scales measure related but distinct constructs. The correlation between the DIF-adjusted community norms scale and the attitudes toward women and men’s relations scale was weak (*r* = 0.087, *p* = 0.018), suggesting that the two scales measure distinct constructs. Table 5 provides the final, validated scale of community norms on domestic labor and household-decision making among male and female youth in Eswatini.

**Table 5:** Final validated single factor four-item domestic labor and household decision-making community norms scale, male and female youth aged 13-24 in Eswatini.

Introduction: I am going to read some statements about relationships between men and women. Please tell me if you strongly agree, agree, neither agree nor disagree, disagree or strongly disagree		
**Item**	**Response Categories**	**Recoded Categories:** **1 = equal community norms, 2 = neutral, 3 = unequal community norms**
Most boys and girls in my community do not share household tasks equally, with girls doing more household tasks than boys	1 = Strongly agree 2 = Agree 3= Neither agree nor disagree 4 = Disagree 5 = Strongly disagree	1 = Disagree OR strongly disagree 2=Neither agree nor disagree 3 = Strongly agree OR agree
Most people in my community expect men to have the final word about decisions in the home		
Most people in my community do not expect girls and boys to share household tasks equally because they expect girls to do more in the household		
Most men in my community are the ones who make the decisions in their home		

## Discussion

Collecting quality data on social norms is essential to inform evidence-based, data-driven policy and practice to address and change norms as drivers of harmful behaviors such as violence [13,48]. However, adolescent populations are often overlooked in measurement of social norms, particularly those related to women and men’s roles, behaviors and responsibilities in society. This study was designed to test the reliability and validity of a novel community norms measure for a national sample of adolescents in Eswatini, to determine if we can capture population-level data on community norms across a broad age range of adolescents, aged 13–24 years. EFA and CFA validation methods yielded a four-item measure capturing both descriptive and injunctive items on community norms related to domestic labor distribution for youth and household decision-making control for adults. The measure shows good reliability and significant correlation with IPV attitudes, which is often used as a proxy for norms measurement [49,50].

Our original items for measurement development included descriptive and injunctive norms related to youth school prioritization, domestic labor responsibilities, early marriage, and parental control over their behavior, as well as norms related to adults’ household decision-making and working outside the home. Endorsement of items was consistent with extant data on women’s and men’s, and boy’s and girl’s lived realities in Eswatini. For example, the majority of youth disagreed that female school participation is tied to completed domestic labor. This finding aligns with parity in schooling attainment between male and female youth in Eswatini, in part due to decades of governmental action to ensure equality in schooling access [31]. Conversely, in Eswatini, where a common siSwati expression, “livi lendvodza ekugcine” (translated to “a final word is uttered by a man”) underscores the embeddedness of norms around men’s decision-making authority [31], most youth agreed that men were the primary decision-makers in the home. Ultimately, only domestic labor distribution for youth and household decision-making control for adults were retained in the final validated measure. Community norms related to education, marriage, and employment were not retained in the final measure. Lack of factor loading for items regarding child marriage may be due to the low rates of this practice in Eswatini [34,51]. Socially normative expectations related to domestic labor responsibilities and decision-making authority may be more salient across country and cultural contexts, regardless of differences between marital age, education, and employment between women and men, girls and boys. Future testing of the full array of items in additional country contexts may be useful.

We also observed differentiation of measurement between age cohorts and sex. Younger compared with older adolescents were more likely to have missing data, respond ‘don’t know’ or decline to answer. This is not likely related solely to use of community norms measures and may reflect limited understanding or awareness of community norms questions among younger adolescents. Alternatively, it could be related to social desirability bias of responses, such that younger adolescents were unsure of the “correct” response and defaulted to a don’t know response. It will be important to monitor age differentiation in survey response in future use of this measure. Findings from this study indicate that the goodness of fit of the measure was better for female participants compared to male participants, though the scale still performed well among males. We also observed differential item functioning on two items by sex. These findings may simply indicate that the norms are more meaningful to girls and young women, as they are the ones marginalized by these norms, with expectations of greater domestic labor load and lesser decision-making authority [31].

Overall, this measure is theory-based, including both injunctive and descriptive norms related to household tasks and decision making, and includes a specific reference group. Given resource constraints and on the ground realities, a shorter scale such as this may be more feasible to measure shifts in community norms over time in large-scale, nationally representative surveys. As demonstrated in this study, a four-item measure could be routinely included in household surveys like VACS, providing critical information to assess and monitor shifts in community norms and their relationships with outcomes of interest.

### Limitations

While this measure offers a useful tool for the field, findings do indicate the need for further study on this topic. First, retained scale items were related only to household tasks and decision making, rather than violence, marriage, school, or work outside the home. Further research should explore how well norms around household tasks and decision-making serve as valid indicators of broader constructs and are associated with outcomes of interest. Further measurement testing is needed for other domains. Second, this study uses only a single country context. A remaining question is whether the measure will be proven valid in other settings. Thus, next steps include circulating the measure for broader use, incorporating, and testing the measure in global surveys, including VACS, and exploring the measure’s relationship with health and behavioral outcomes. Further analysis by sex, age and other socio-demographic characteristics would also be fruitful. Future investigation into how youth of varied age groups understand and report on social norms is critical given that perceptions of social norms likely change dramatically between the ages of 13 and 24 as youth traverse major developmental changes. Finally, we assessed construct validity using only two attitudes scale. Future research should evaluate how this community norms measure is associated with other conceptually relevant constructs, including peer and parental interpersonal relationships, witnessing violence in the household and community, exposure to childhood violence before age 18, and perpetration of intimate partner violence in early adulthood.

## Conclusion

This study resulted in a promising single factor four-item community norms scale. It meets the requirement of a brief, theory-based measure, feasible to incorporate into national and cross- national surveys. In addition to providing a much-needed community norms measure, capturing household decision-making and domestic labor, for use in large scale surveys, this analysis revealed actionable information about community norms in Eswatini. Results show that youth perceive their communities to be more equal between boys and girls with respect to education, and less so when it comes to decision-making power, suggesting that policy and program efforts may need to prioritize barriers to girls’ education. Efforts to address unequal norms between women and men, boys and girls, might be more effectively focused on decision-making power within the household.

## Supporting information

S1 Table Unweighted descriptives of original response categories for Eswatini Violence Against Children and Youth Survey (VACS) novel community norms measure (n = 7,720). (PDF)

S2 Table Exploratory and confirmatory factor analysis of the preliminary 2-factor community norms measures among youth ages 13–24 years, 2022 Eswatini Violence Against Children and Youth Survey. (PDF)
