# Supplementary material for: Psychometric validation of a novel community norms measure among youth, Eswatini Violence Against Children and Youth Survey, 2022
Source: PLoS One. 2026 May 20;21(5):e0345048. doi: 10.1371/journal.pone.0345048 (PMC13189306; doi:10.1371/journal.pone.0345048)
Supplement: S1 Table — (PDF) [file pone.0345048.s001.pdf]

Supplementary Table 1. Unweighted descriptives of original response categories for Eswatini Violence Against Children and Youth Survey (VACS) novel community norms measure (n=7,720)

Item	Item Description		Strongly agree	Agree	Neither agree/ disagree	Disagree	Strong Disagree	Don't Know	Decline
1	Our culture makes it harder for girls to achieve their goals than boys	n	666	2,730	717	2,602	256	743	6
		%	8.63	35.36	9.29	33.7	3.32	9.62	0.08
2	Adolescent girls in my community are more likely to be out of school than adolescent boys	n	262	2,153	667	3,693	521	420	4
		%	3.39	27.89	8.64	47.84	6.75	5.44	0.05
3	Girls in my community are sent to school only if they are not needed to help at home	n	47	584	492	5,154	1,081	356	6
		%	0.61	7.56	6.37	66.76	14	4.61	0.08
4	Most people in my community expect girls to be sent to school only if they are not needed at home	n	59	597	486	5,046	1,153	372	7
		%	0.76	7.73	6.3	65.36	14.94	4.82	0.09
5	Most boys and girls in my community do not share household tasks equally, with girls doing more household tasks than boys	n	969	3,699	484	1,921	187	455	5
		%	12.55	47.91	6.27	24.88	2.42	5.89	0.06
6	Most people in my community expect men to have the final word about decisions in the home	n	1,194	3,816	519	1,548	125	516	2
		%	15.47	49.43	6.72	20.05	1.62	6.68	0.03
7	Most people in my community do not expect girls and boys to share household tasks equally because they expect girls to do more in the household	n	759	3,259	575	2,323	270	530	4
		%	9.83	42.22	7.45	30.09	3.5	6.87	0.05
8	Most men in my community are the ones who make the decisions in their home	n	1,674	4,189	453	825	81	495	3
		%	21.68	54.26	5.87	10.69	1.05	6.41	0.04
9	Women in my community work outside the home	n	618	4,331	458	1,261	89	961	2
		%	8.01	56.1	5.93	16.33	1.15	12.45	0.03
10	Most people in my community believe that women should be able to work outside the home if they want	n	558	4,212	547	1,165	106	1,132	0
		%	7.23	54.56	7.09	15.09	1.37	14.66	0
11	Most adolescent girls in my community marry before the age of 18 years	n	75	772	374	4,717	1,067	713	2
		%	0.97	10	4.84	61.1	13.82	9.24	0.03
12	Adults in my community expect adolescent girls to get married before the age of 18 years	n	44	533	431	4,604	1,320	786	2
		%	0.57	6.9	5.58	59.64	17.1	10.18	0.03
13		n	1,262	3,794	500	1,409	120	630	5

	Most families in my community control their daughters' behaviors more than their sons'	%	16.35	49.15	6.48	18.25	1.55	8.16	0.06
14	Most people in my community expect families to control their daughter's behavior more than their sons'	n	1,431	4,042	481	1,039	104	620	3
		%	18.54	52.36	6.23	13.46	1.35	8.03	0.04
Omitted from analysis*	Of the married men or men in relationships in your community, how many would you say hit their wives or girlfriends/romantic partners to correct them when they feel the wife/girlfriend/romantic partner has done something wrong?	n	43	232	115	1,165	2,960	3,162	3
		%	0.56	3.01	2.01	15.09	38,34	40.96	0.04
15	Most adults in my community intervene, for example, talk to the family or call police or other authorities, if their neighbor is hitting or intentionally hurting, his wife.	n	697	3,350	369	690	115	2,499	0
		%	9.03	43.39	4.78	8.94	1.49	32.37	0
16	Most adults in my community intervene, for example, talk to the family or call police or other authorities, if their neighbor is hitting or intentionally hurting, their child.	n	669	3,352	345	869	157	2,328	0
		%	8.67	43.42	4.47	11.26	2.03	30.16	0

*Note: *Response options for item GN19 are: almost all of the men in the community, more than half of the men in the community, less than half of the men in the community, no men in the community, don't know, declined. Item omitted from analysis due to >40% missingness.*
