# Supplementary material for: Psychometric validation of a novel community norms measure among youth, Eswatini Violence Against Children and Youth Survey, 2022
Source: PLoS One. 2026 May 20;21(5):e0345048. doi: 10.1371/journal.pone.0345048 (PMC13189306; doi:10.1371/journal.pone.0345048)
Supplement: S2 Table — (PDF) [file pone.0345048.s002.pdf]

**Supplementary Table 2. Exploratory and confirmatory factor analysis of the preliminary 2-factor community norms measures among youth ages 13-24 years, 2022 Eswatini Violence Against Children and Youth Survey**

| | EFA (n=3,842)± | CFA (n=3,827) ± | | |
|---|---|---|---|---|
| | **Loading** | **Est.** | **S.E.** | ***p*** |
| Factor 1 | | | | |
| Item 2. Adolescent girls in my community are more likely to be out of school than adolescent boys | 0.512 | 0.617 | 0.038 | <0.001 |
| Item 3. Girls in my community are sent to school only if they are not needed to help at home | 0.945 | 0.951 | 0.033 | <0.001 |
| Item 4. Most people in my community expect girls to be sent to school only if they are not needed at home | 0.944 | 0.938 | 0.031 | <0.001 |
| Factor 2 | | | | |
| Item 5. Most boys and girls in my community do not share household tasks equally, with girls doing more household tasks than boys | 0.667 | 0.713 | 0.029 | <0.001 |
| Item 6. Most people in my community expect men to have the final word about decisions in the home | 0.785 | 0.775 | 0.030 | <0.001 |
| Item 7. Most people in my community do not expect girls and boys to share household tasks equally because they expect girls to do more in the household | 0.745 | 0.763 | 0.025 | <0.001 |
| Item 8. Most men in my community are the ones who make the decisions in their home | 0.731 | 0.716 | 0.033 | <0.001 |
| Correlation, F1 F2 | - | 0.176 | 0.063 | 0.005 |
| RMSEA (90% Confidence Interval) | 0.028 | 0.061 | | |
| CFI | 0.990 | 0.934 | | |
| TLI | 0.975 | 0.894 | | |
| SRMR | 0.041 | 0.096 | | |

*Significant at the .05 level. ±The full analytic sample (n = 7,709) was randomly split into two halves for EFA and CFA validation.
